# Extrinsic stabilization of antiviral ACE2-Fc fusion proteins targeting SARS-CoV-2

**DOI:** 10.1038/s42003-023-04762-w

**Published:** 2023-04-08

**Authors:** Hristo L. Svilenov, Florent Delhommel, Till Siebenmorgen, Florian Rührnößl, Grzegorz M. Popowicz, Alwin Reiter, Michael Sattler, Carsten Brockmeyer, Johannes Buchner

**Affiliations:** 1 Center for Functional Protein Assemblies (CPA) and School of Natural Sciences, Department of Bioscience, Technical University of Munich, 85748 Garching, Germany; 2 Institute of Structural Biology, Helmholtz Zentrum München, Neuherberg, Germany; 3 Bavarian NMR Center, School of Natural Sciences, Department of Bioscience, Technical University of Munich, Garching, 85748 Munich Germany; 4 Formycon AG, Martinsried/Planegg, Germany; 5 Brockmeyer Biopharma GmbH, Senator-Ernst Str. 2, Marzling, Germany; 6 Present Address: Faculty of Pharmaceutical Sciences, Ghent University, Ottergemsesteenweg 460, 9000 Ghent, Belgium

**Keywords:** Recombinant protein therapy, Recombinant protein therapy

## Abstract

The angiotensin-converting enzyme 2 (ACE2) is a viral receptor used by sarbecoviruses to infect cells. Fusion proteins comprising extracellular ACE2 domains and the Fc part of immunoglobulins exhibit high virus neutralization efficiency, but the structure and stability of these molecules are poorly understood. We show that although the hinge between the ACE2 and the IgG4-Fc is highly flexible, the conformational dynamics of the two ACE2 domains is restricted by their association. Interestingly, the conformational stability of the ACE2 moiety is much lower than that of the Fc part. We found that chemical compounds binding to ACE2, such as DX600 and MLN4760, can be used to strongly increase the thermal stability of the ACE2 by different mechanisms. Together, our findings reveal a general concept for stabilizing the labile receptor segments of therapeutic antiviral fusion proteins by chemical compounds.

## Abstract (web summary)

The solution structure, stability, and dynamics of a broadly-acting antiviral ACE2-IgG-Fc fusion protein are determined. Small chemical compounds binding to ACE2 can be used to drastically increase the thermal stability of the ACE2 domain.

## Introduction

The COVID-19 pandemic revealed our vulnerability towards emerging viruses. Universal antivirals are urgently needed to fight novel variants of SARS-CoV-2 and to control future outbreaks^1,2^. Virus entry inhibitors employing the host protein to which the viruses attach are a promising strategy in this context.

Sarbecoviruses, a subfamily of betacoronaviruses, including the severe acute respiratory syndrome coronavirus 2 (SARS-CoV-2) use the angiotensin-converting enzyme 2 (ACE2) to infect cells^3^. ACE2 is a transmembrane protein composed of 805 residues organized into extracellular, transmembrane and cytosolic domains^4^. The extracellular part of ACE2 contains a protease domain (PD) and a collectrin-like domain (CLD)^5^. The PD consists of two subdomains which form the catalytic site required to cleave angiotensin II to angiotensin (1–7)^4^, thus regulating the renin-angiotensin-aldosterone system (RAAS)^6^.

Because the binding of the virus to ACE2 is pivotal for the viral infection, strategies to fight COVID-19 infections have been developed that target this interaction^7^. Several monoclonal antibodies (mAbs) binding the receptor-binding domain (RBD) of SARS-CoV-2 have entered the clinics^8^. However, these antibodies are often effective only against a specific virus strain while newly emerging variants of concern (VoC), such as the Omicron variant of SARS-CoV-2, which exhibit multiple mutations in the viral spike protein, often decrease their activities^9,10^.

**Fig. 1:**
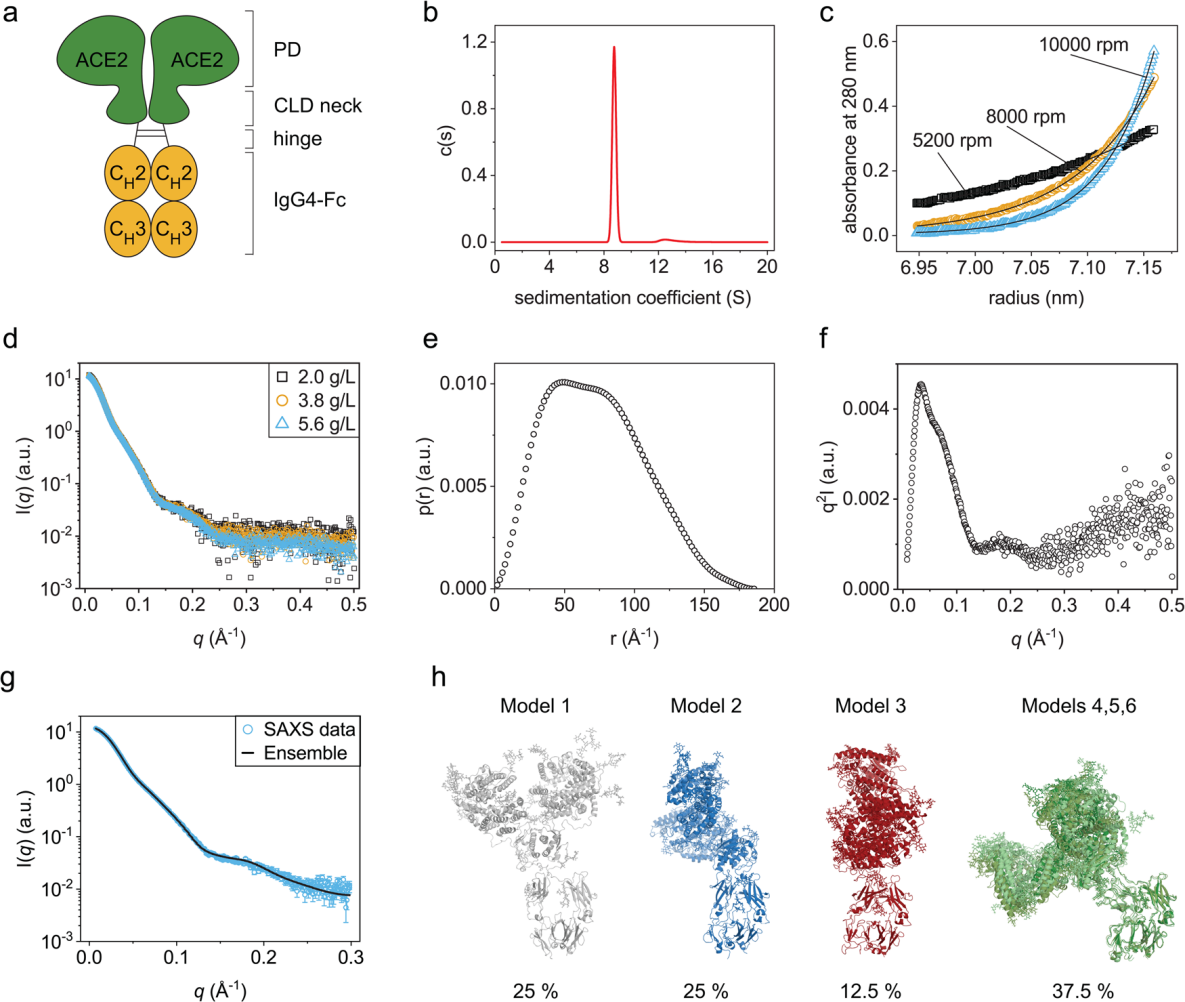
Solution structure of ACE2-IgG4-Fc. **a** Schematic structure of ACE2-IgG4-Fc. **b** AUC-SV measurement showing a monodisperse sample. **c** AUC-SE measurement. The black lines are fits to the data from which the molecular mass was determined in Sedfit. **d** SAXS data obtained with different ACE2-IgG4-Fc concentrations. **e** Pairwise distribution from the analysis of the SAXS data. **f** Kratky plot. **g** Fit of the back-calculated SAXS curves of the best structural ensemble overlayed with the experimental data. **h** Overview of the selected models in the conformational ensemble with their respective populations. Structures are superimposed with respect to the IgG4-Fc domains. Models 4, 5, 6 show a very similar conformation and consequently are shown overlayed together on the figure.

A promising alternative therapeutic strategy is to use viral receptors as traps that neutralize the virus^11^. A soluble form of ACE2 has been successfully used in a clinical setting^12^. To prolong the serum half-life and link it to the immune system, the ACE2 segment can be fused to the constant Fc-part from human immunoglobulin G (IgG) subclasses^13–17^ (Fig. 1a). These ACE2-Fc molecules exhibited increased potency to neutralize infectious SARS-CoV-2 VoCs^17^, including the Omicron variant^13,18,19^. The promising results obtained suggest that ACE2-Fc fusions are antiviral molecules that have the potential to become universal drugs against viruses that employ the ACE2 receptor to enter cells. However, still little is known about the ACE2-Fc structure, dynamics and stability. Here we defined the solution structure of a proto-typic ACE2-Fc fusion protein. We found that the ACE2 part exhibits strongly decreased stability compared to the Fc moiety. Notably, chemical compounds binding to ACE2 can be used to increase its conformational stability by distinct allosteric effects.

## Results

### The ACE2-IgG4-Fc is a monodisperse homodimer with the ACE2 domains in an open conformation

An ACE2-IgG4-Fc fusion protein had been designed as a universal protein therapeutic against viruses using the human ACE2 receptor to infect cells^17^. The protein contains a wild-type human ACE2 domain (residues Q18-G732) and a human IgG4-Fc part (Fig. 1a) with a stabilizing hinge mutation (S228P)^20^.

Our preliminary analysis had shown that the ACE2-IgG4-Fc is a homodimer^17^ (Fig. 1a). However, an important open question was the extent to which the two ACE2 domains in the fusion protein dimer are associated with each other. In both reported structures of the full-length ACE2 dimer^21^, the neck domains of the CLDs dimerize. In the closed conformation, the two protease domains (PDs) also contact each other, while no direct contacts between the PDs are observed in the open conformation, although the two domains are still close to each other. Here we aimed to characterize the structure and conformational dynamics of ACE2-Fc in solution. Analytical ultracentrifugation sedimentation velocity measurements (AUC-SV) revealed a narrow peak with a sedimentation coefficient of 8.8 S and a frictional ratio *f/f*_*0*_ = 1.62 ± 0.03 (Fig. 1b) indicating that ACE2-Fc is highly monodisperse with a shape similar to immunoglobulin G (IgG) antibodies^22^. A sedimentation equilibrium (AUC-SE) analysis yielded an *M*_*m*_ of 258 ± 2.4 kDa which is close to the mass of an ACE2-Fc homodimer determined from the amino acid sequence (217 kDa) plus the mass of the glycans which can be around 30 kDa^23^. Therefore, within a reasonable experimental error, the *M*_*m*_ from the AUC-SE experiments corresponds well to a glycosylated homodimer (Fig. 1c).

After confirming that ACE2-Fc is monodisperse, we used small-angle X-ray scattering (SAXS) to obtain further insights into the structure and shape of the fusion protein in solution. The SAXS curves at small angles obtained for different protein concentrations are superimposable (Fig. 1d), indicating the absence of aggregation. The calculated radius of gyration (*R*_*g*_) from the Guinier analysis is 55 Å. The pair distance distribution function shows two maxima and a maximum pairwise distance, *D*_max_, of ~185 Å (Fig. 1e), consistent with the maximum distance expected for the dimeric fusion protein (see below). Finally, the Kratky plot is characteristic of a globular multi-domain protein (Fig. 1f). These observations are consistent with the hypothesis that ACE2-Fc is a homodimer with a slightly larger size compared to IgGs^24^.

To characterize the conformation and flexibility of ACE2-IgG4-Fc, we performed molecular dynamics (MD) simulations of the fusion protein and evaluated the conformational ensemble by comparing with the experimental SAXS data. We computationally generated 6000 conformations of glycosylated ACE2-IgG4-Fc based on the available open and closed ACE2s structures^21^. ACE2-IgG4-Fc is expected to adopt multiple conformations in solution due to the flexible linker connecting the Fc and ACE2 parts. Thus, a genetic algorithm was used to define ensembles of conformations, for which the ensemble-average back-calculated SAXS profile fitted best with the experimental data. An ensemble of 6 structures was selected and showed a very good fit with a χ^2^ of 1.7 to the SAXS data (Fig. 1g). Within the selected ensemble, the main divergence originates from the differential orientation of the ACE2 region and the IgG4-Fc region (Fig. 1h). This suggests a high level of flexibility of the linker connecting the two domains. Of note, the distance between the center of mass (COM) of the PD domains (defined as residues 18–601) within the dimers also shows some variability between models from 59 to 81 Å and an average value of 68 Å (Supplementary Fig. 1). The structure of ACE2 in complex with B^0^AT1 was solved in two distinct conformations, a so-called closed state, in which the PD domains make direct contact and an open state in which no interaction is observed between the PDs. Consequently, these two states exhibit a slightly different COM distance, corresponding to 64 Å and 67 Å, for the closed and open states, respectively. The average COM distance value from our ensemble is closer to the open state, suggesting that this is a preferred conformation in solution. However, the variability of our ensemble and the relative small difference observed between states does not allow to exclude the presence of closed state conformations in solution (Supplementary Fig. 1).

### The ACE2 domain is less stable and unfolds independently of the Fc

**Fig. 2:**
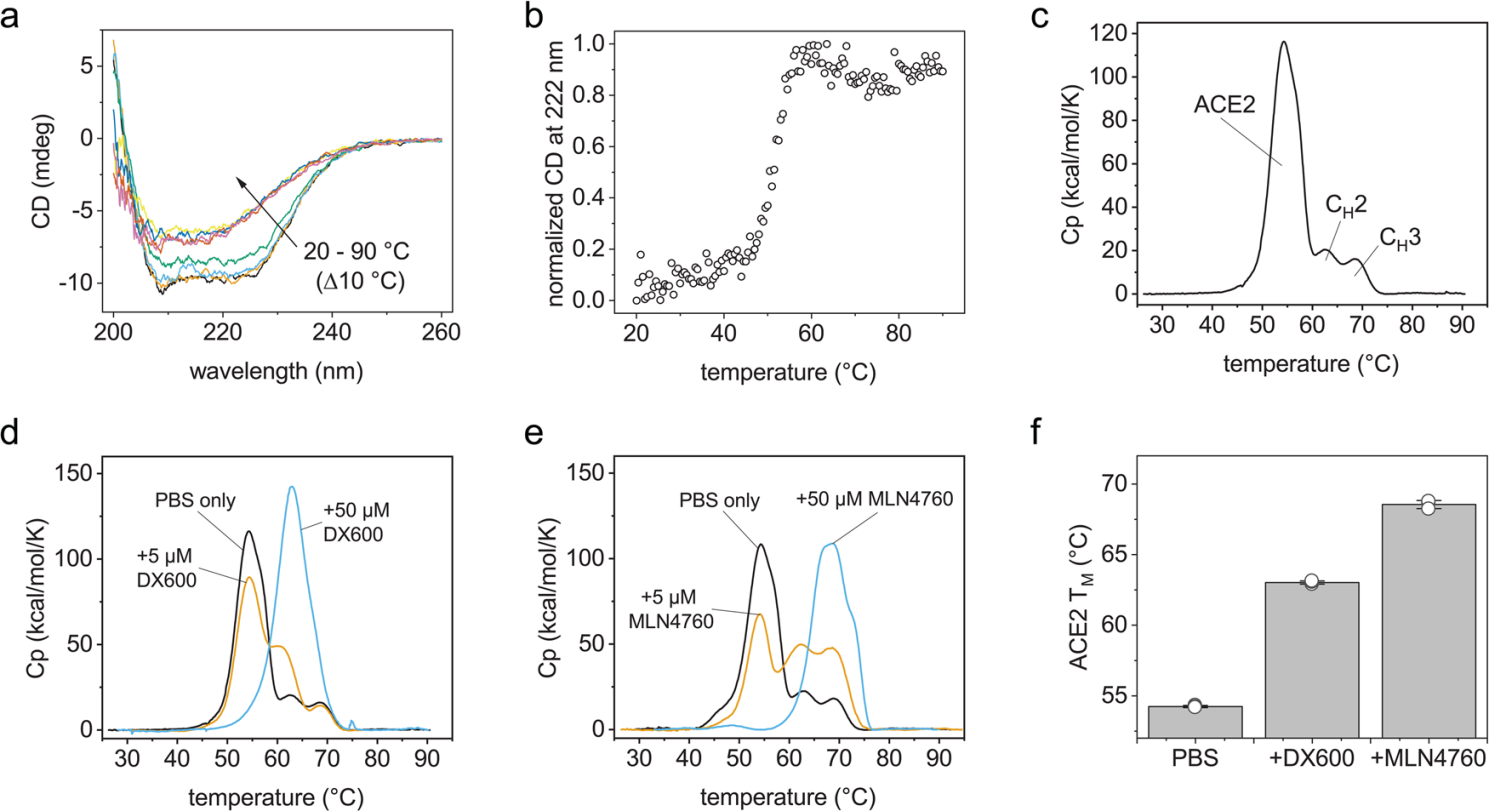
Thermal stability of ACE2-IgG4-Fc. **a** FUV CD spectra obtained at different temperatures. **b** Change in the CD signal at 222 nm during heating. DSC thermograms of ACE2-IgG4-Fc (**c**) without inhibitors or in the presence of (**d**) DX600 or (**e**) MLN4760. The concentration of ACE2-IgG4-Fc was 5 µM. **f** Melting temperatures of the ACE2 domain in ACE2-IgG4-Fc (5 µM) without inhibitor or with 50 µM inhibitor. The bars are mean values of triplicates. The error bar is the standard deviation. The circles depict the values from each replicate.

After elucidating the solution structure and hinge flexibility in ACE2-IgG4-Fc, we asked whether the fusion protein is conformationally stable and whether the Fc domain affects the stability of the ACE2. When we monitored the thermal stability of the ACE2-IgG4-Fc by circular dichroism (CD) spectroscopy, an irreversible shift of the CD spectra was observed above 50 °C (Fig. 2a) with a T_M_ = 50.9 ± 0.1 °C (Fig. 2b). To gain more insight into stability of the individual parts of the fusion protein, differential scanning microcalorimetry (DSC) was employed. Here, three distinct unfolding events could be resolved and assigned to the different domains in ACE2-IgG4-Fc (Fig. 2c). The T_M_s were 54.2 ± 0.1 °C, 62.8 ± 0.1 °C and 68.6 ± 0.2 °C for ACE2, C_H_2 and C_H_3, respectively. Thus, the ACE2 is the thermodynamically least stable domain of the fusion protein. To investigate whether the Fc partner affects the stability of the ACE2, we also analyzed an ACE2-IgG1-Fc protein. As expected, the C_H_2 and C_H_3 domains from the IgG1 showed different melting temperatures compared to IgG4 (Supplementary Fig. 2), but the T_M_ of the ACE2 domain in the ACE2-IgG1-Fc was around 54 °C just as the ACE2 T_M_ measured for the ACE2-IgG4-Fc. Taken together, the structural and stability analysis indicates that the conformational stability of ACE2 could be limiting in most ACE2-fusion proteins. Therefore, strategies to stabilize the ACE2 domain will be highly valuable.

### The ACE2 domain is stabilized by enzyme inhibitors

We wondered whether it would be possible to extrinsically stabilize the ACE2 domains in the fusion protein by the addition of chemical compounds. As low molecular weight ligands such as enzyme inhibitors can increase the stability of catalytic domains^25^, we considered potent inhibitors of ACE2 activity. The ACE inhibitor MLN4760 inserts into the enzymatic cleft of ACE2 and causes structural rearrangements in the PD^26^. A second inhibitor is a 3-kD peptide (DX600) with a random coil structure^27,28^. Both MLN4760 and DX600 inhibit the enzymatic activity of ACE2 with inhibitory concentrations in the low nanomolar range^28,29^. Both molecules, MLN4760 and DX600, had been reported to inhibit the enzymatic activity of ACE2^28,29^. When the inhibitors were added in ~1:1 molar ratio, the unfolding of some of the ACE2 domains was shifted to higher temperatures (Fig. 2d, e). For shifting the entire unfolding peak of the ACE to higher temperatures a 10:1 molar excess of the inhibitors was required (Fig. 2d, e). For DX600 the T_M_ of the ACE2 domain was increased by 8.8 °C (Fig. 2f) and the stabilization was even more pronounced (14.3 °C) when MLN4760 was present in a 10-fold molar excess (Fig. 2f). Interestingly, the stabilizing effects of the two inhibitors were not additive (Supplementary Fig. 3).

### The binding mechanisms of the ACE2 inhibitors are different

**Fig. 3:**
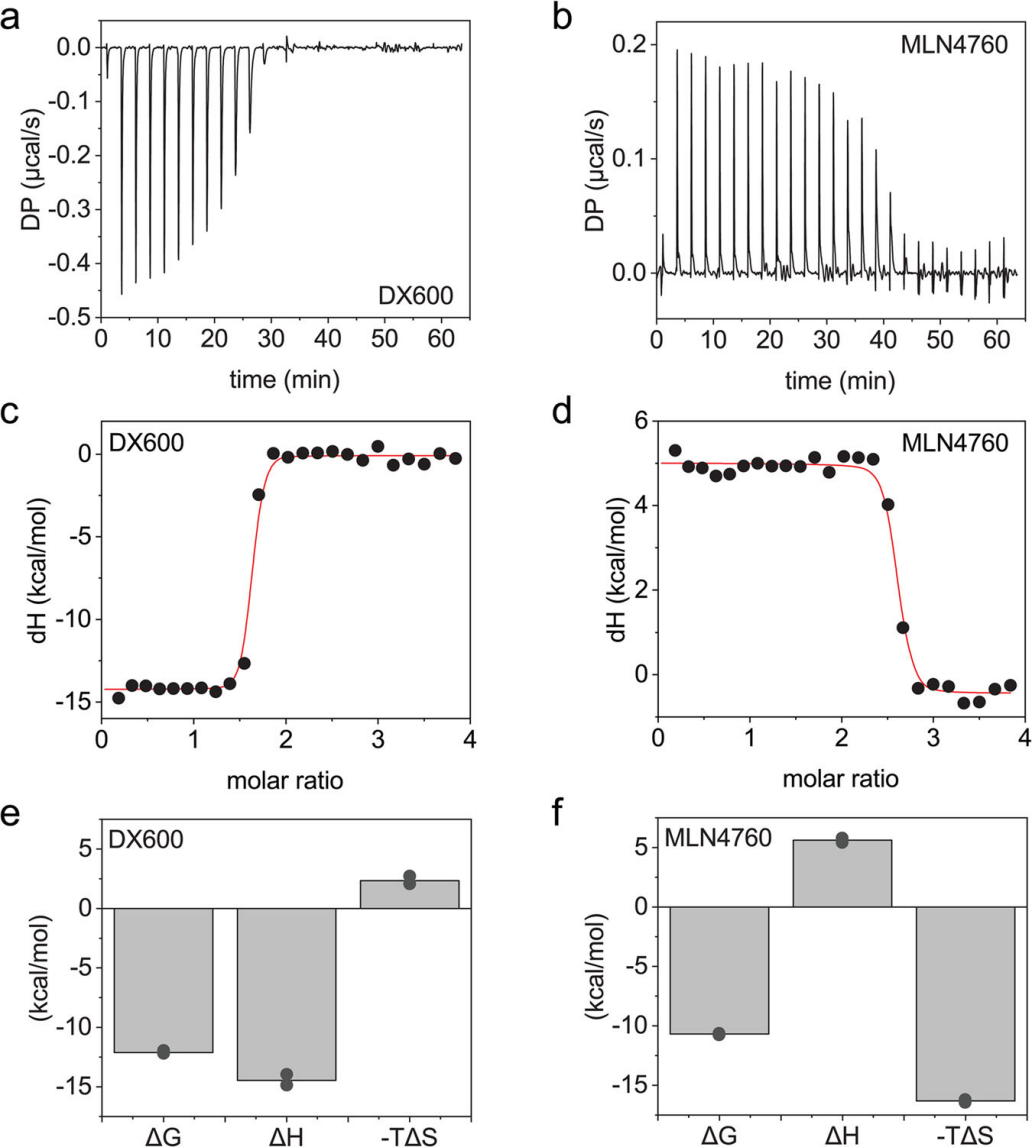
Binding of enzyme inhibitors to ACE2-IgG4-Fc. ITC measurements to assess the binding of (**a**) DX600 and (**b**) MLN4760 to ACE2-IgG4-Fc. Fits to the ITC data obtained with (**c**) DX600 and (**d**) MLN4760. The red lines are fits to the experimental data. Thermodynamic parameters of the binding of (**e**) DX600 and (**f**) MLN4760 to ACE2-IgG4-Fc. The bars are mean values of duplicates. The values from each replicate are shown in circles.

Since we observed different degrees of ACE2 stabilization by DX600 and MLN4760, we further investigated the interaction between the inhibitors and the fusion protein. To this end, we used isothermal titration calorimetry (ITC) (Fig. 3a–f). The binding constants (dissociation constant, *K*_D_) for the interactions are in the low nanomolar range (1.3 ± 0.3 nM for DX600 and 8.1 ± 1.3 nM for MLN4760). The stoichiometry is fitted to ≈1.5 for DX600 and 2.5 for MLN4760, consistent with the presence of two enzymatic sites in the ACE2-IgG4-Fc. Strikingly, the enthalpic component of the interaction is negative for DX600 but positive for MLN4760 hinting towards different binding mechanisms employed by the two inhibitors (Fig. 3e, f).

### The enzyme inhibitors do not affect the binding of ACE2-Fc to the viral spike protein

**Fig. 4:**
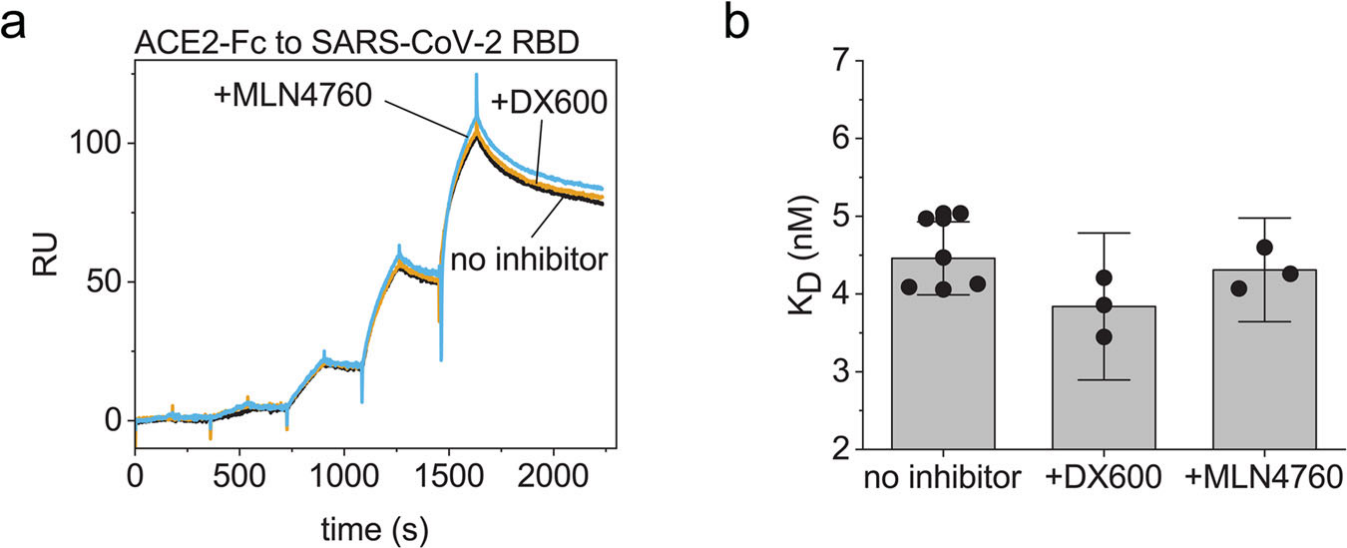
Binding of ACE2-IgG4-Fc to immobilized SARS-CoV-2 RBD. **a** Overlay of sensorgrams. **b** Calculated binding constants from the measurements. The bar shows the mean value with 95% confidence intervals as error bars. The circles are the replicates.

We were interested whether the strong interaction between the inhibitors and the ACE2 domain affects the binding of ACE2-IgG4-Fc to the RBD of SARS-CoV-2. We used surface plasmon resonance (SPR) to analyze the affinity of ACE2-Fc to the immobilized RBD without inhibitors or in 10:1 molar excess of the inhibitor. The SPR sensorgrams obtained with or without inhibitors were superimposable (Fig. 4a). The calculated affinities for the ACE2-IgG4-Fc binding to the RBD are *K*_*D*_ ≈ 4 nM (Fig. 4b).

### The two enzyme inhibitors have different effects on the structural dynamics of ACE2-Fc

**Fig. 5:**
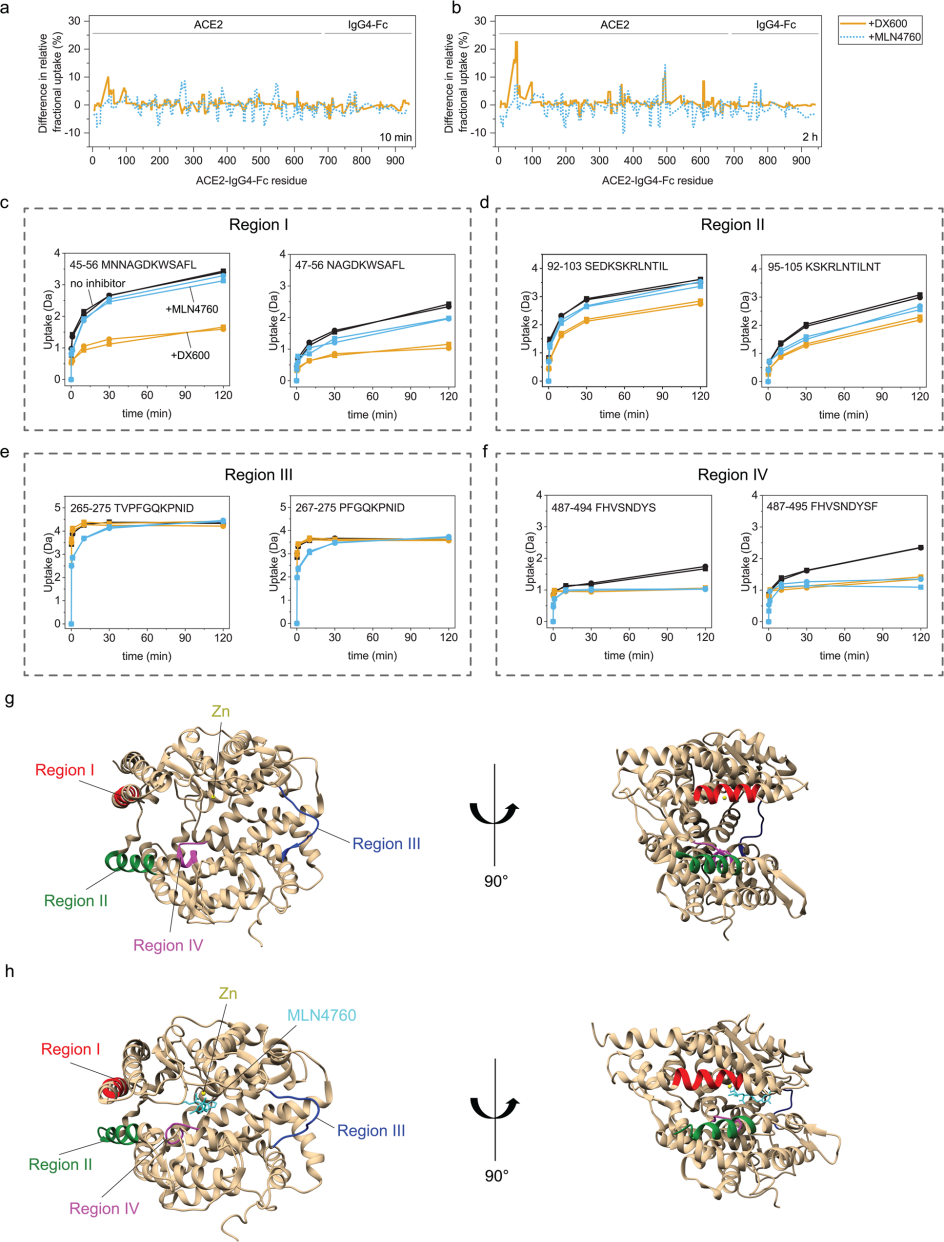
Changes in the structural dynamics of ACE2-IgG4-Fc caused by the inhibitors. Difference in the relative fractional uptake from HDX measurements obtained at (**a**) 10 min and (**b**) 2 h exchange times. The plots show differences in the relative fractional uptake of ACE2-Fc without an inhibitor minus the relative fractional uptake of inhibitor-bound ACE2-Fc. (**c**–**f**) Deuterium uptake plots for the peptides in four identified regions. Crystal structure of (**g**) inhibitor-free ACE2 (PDB: 1R42) and (**h**) the ACE2 bound to MLN4760 (PDB: 1R4L) with annotations of the regions identified by HDX.

To further elucidate the dynamics of the fusion protein and structural features that mediate the stabilization of ACE2-IgG4-Fc by the inhibitors, we used hydrogen-deuterium exchange (HDX) coupled to mass spectrometry (MS). HDX revealed that that the inhibitors had minor effect on the constant Fc part but considerable influence on the ACE2 domains. Strikingly, the two inhibitors affected the HDX in distinct parts of the ACE2 domain (Fig. 5a, b). We identified several peptide regions exhibiting less deuterium uptake in the presence of the inhibitors. In regions I and II, DX600 reduced the uptake more than MLN4760 (Fig. 5c, d). In region III, only MLN4760 reduced the deuterium uptake in the first 30 min of the HDX reaction (Fig. 5e). In region IV, both inhibitors exhibited effects (Fig. 5f). The HDX were mapped on the crystal structure of ACE2 to visualize the position of the identified regions^26^ (Fig. 5g). Regions I and II are in two alpha helices on the surface of ACE2. Region III is at the hinge region between the two subdomains of the PD that form a cleft. Region IV is a loop close to the enzymatic site. By comparing the inhibitor-free to the MLN4760-bound ACE2, substantial structural changes leading to a closer positioning of the two subdomains in the PD can be observed (Fig. 5g, h)^26^. Noteworthily, MLN4760 but not DX600 affects the HDX in other peptides along the axis of subdomain movement (Supplementary Fig. 4). Taken together the HDX and the structural data imply that the binding of MLN4760 has stronger allosteric effects on ACE2 compared to DX600.

## Discussion

Potent and universal antiviral therapeutics are urgently needed. We have previously shown that ACE2-Fc constructs exhibit high neutralization efficiency against sarbecoviruses^17^. Similar findings were also reported for other ACE2-Fc molecules^13–16,18,19^. ACE2-Fcs are therefore emerging as promising candidates against viruses employing the ACE2 receptor. However, a better understanding of the structure and stability of these fusion proteins is required. In this study, we analyzed the solution structure of an ACE2-IgG4-Fc and show that the conformational stability of the ACE2-domain is lower than that of the Fc-moiety. This can be ameliorated by extrinsic factors which bind to ACE2 and stabilize the domain by different mechanisms.

Our results show that the ACE2-IgG4-Fc exists as a monodisperse homodimer in solution. The structural analysis combing SAXS and MD simulations revealed that the Fc hinge region is highly flexible and is thereby responsible for the heterogeneity concerning the relative orientations of the ACE2 and Fc parts of the ACE2-IgG4-Fc protein in solution. Our data also suggest that the ACE2 domains are in a preferential open conformation similar to reported structures of full-length ACE2 dimers^21^, while a minor population of closed ACE2 dimer conformations is likely also present. This finding is interesting because the proximity of the ACE2 domains in ACE2-IgG4-Fc was expected to facilitate dimerization of the two PDs based on crystal structures. However, the PD dimerization was observed in full-length ACE2 dimers in quaternary complex with the neutral amino acid transporter B^0^AT1^21^. It is possible that the transmembrane domain of ACE2 (which is lacking in ACE2-Fc) or B^0^AT1 play a role for the dimerization of the PDs.

Sufficiently high stability is a prerequisite for the development of fusion proteins as biotherapeutics. The thermodynamic stability of the ACE2 domain is significantly lower compared to the Fc part as evident from the DSC measurements. In addition, the thermodynamic stability of the isolated ACE2 ectodomain is identical that of the ACE2 domain in the context of the fusion with an Fc fragment^30^. We therefore asked whether we could stabilize the protein without introducing mutations. We observed a substantial stabilization of ACE2 in the presence of two enzyme ACE2 inhibitors. Despite similar affinity, MLN4760 was more potent than DX600, hinting towards different stabilization mechanisms. Our ITC analysis supported this hypothesis by revealing different binding mechanisms for the two enzyme inhibitors. The binding between DX600 and ACE2-IgG4-Fc is enthalpically driven, while MLN4760 binds with a favourable entropic component. The enthalpy-driven binding suggests the formation of hydrogen bonds between the peptide DX600 and the ACE2 domain^31^. In the case of MLN4760, hydrophobic interaction or structural rearrangements of the ACE2 domain could explain the strong entropic contribution^31,32^. The hypothesis of hydrophobic interactions agrees well with a published crystal structure where MLN4760 is embedded into the hydrophobic environment inside the enzymatic cleft^26^. In addition, the binding constants that we determined with ITC agree with the published inhibitory concentrations^28,29^.

Consistent with the proposed different binding mechanisms, we observed that the two inhibitors affect the HDX in different regions of the ACE2. The peptide DX600 reduces the deuterium uptake in two alpha-helices on the outer surface of the ACE2 domain. The binding of DX600 to this region via hydrogen bonds would explain the enthalpy-driven interaction that we observed in ITC.

In contrast, MLN4760 had less influence on the HDX in the α-helices of ACE2 but reduced the deuterium uptake in different regions compared to DX600. For example, region III (residues 265–275) was affected by MLN4760 but not by DX600. Interestingly, residues 265–275 are in the hinge region between the two subdomains of ACE2’s PD. The hinge region bends upon binding of MLN4760 causing a shift of the relative orientation between the two subdomains of about 16°^26^. Other residues along the axis of the hinge between the PD domains are 81–82, 378–379, 391–392, 415–416, 521–530 and 546–550 (according to the numbering that we used for the HDX data). Although the sequence coverage did not allow us to inspect all these regions, peptides encompassing residues 375–385 and 544–541 also showed reduced deuterium uptake in the presence of MLN4760 but not in the presence of DX600 (Supplementary Fig. 4). Taken together, the data suggest that only MLN4760 induces substantial structural changes in ACE2 such as the movement of the hinge between the two subdomains in ACE2 PD. These conformational differences seem to be correlated to the stronger stabilization of the ACE2 domain by MLN4760 compared to DX600 which we observed in the DSC experiments.

Interestingly, neither of the two enzyme inhibitors affected the binding of ACE2-IgG4-Fc to the RBD of SARS-CoV-2. The interaction with the RBD affected the deuterium uptake in ACE2 residues 7–23, 45–55 and 329–340 according to the numbering in our HDX analysis^33^. The DX600 peptide also influenced the deuterium uptake of the helix encompassing residues 45–55 without obstructing the interaction of RBD and ACE2. These findings indicate that the inhibitors could be employed as chemical compounds that turn off the enzymatic activity and stabilize ACE2-fusion proteins without affecting their potency. Both MLN4760 and DX600 were developed as inhibitors of ACE2. In the future, it will be interesting to see whether the ACE2-Fc-bound inhibitors affect the activity of endogenous ACE2 after administration in vivo. Theoretically, if an inhibitor molecule is transferred from ACE2-Fc to an endogenous ACE2, the freed ACE2-Fc should exhibit enzymatic activity that will compensate for the loss enzymatic activity from the endogenous ACE2 occupied by the inhibitor and maintain the normal function of the RAAS. However, animal studies will be needed to ultimately test this hypothesis. Overall, employing chemical compounds that stabilize the ACE2 domain seems a promising strategy to improve the intrinsic stability and inhibit the enzymatic activity of ACE2-Fc fusion proteins at the same time.

## Materials and methods

### Protein sequences and inhibitors

The ACE2-Fc sequences were designed as previously described^17^. Briefly, the ACE2 part included residues Q18-G732. The Fc from IgG4 had a stabilizing mutation (S228P) in the hinge region. For comparison, the same ACE2 sequence variants were fused to the Fc fragment of IgG1 with a truncated hinge region (DKTHTCPPCPA). The ACE2-IgG4-Fc was produced in Chinese hamster ovary (CHO) cells. The ACE2-IgG1-Fc was obtained from HEK293 cells as described^17^. The proteins were purified to homogeneity and their identity and purity were verified by mass spectrometry, gel electrophoresis and size-exclusion chromatography. The measurements of ACE2-Fc were performed in 50 mM tris und 150 mM NaCl (pH 7.5) unless otherwise indicated in the method. The inhibitors, MLN4760 (MilliporeSigma, Cat. Nr. 5.30616.0001) and DX-600 (Selleckchem, Cat. Nr. S9666) were reconstituted according to the protocols by the manufacturers.

### Analytical ultracentrifugation (AUC)

AUC measurements were performed on an Optima™ AUC (Beckman, Krefeld, Germany) equipped with absorbance detector and an AN50-ti rotor. The samples were filled in measurement cells with sapphire glasses and 2-channel Epon charcoal-filled centerpieces. The measurements were performed at 20 °C. The sedimentation velocity (SV) runs were performed at 42,000 rpm. The speeds for the sedimentation equilibrium (SE) experiments were 5200 rpm, 8000 rpm and 10,000 rpm. The centrifugation time was 24 h at each speed. The ACE2-IgG4-Fc concentration was 0.1 and 0.2 mg/mL for the SE and SV experiments, respectively. The AUC data was evaluated in Sedfit v16.1.

### Small-angle X-ray scattering (SAXS)

SAXS data were recorded on a Rigaku BioSAXS1000 instrument with a Rigaku HF007 microfocus rotating anode with a copper target (40 kV, 30 mA). Transmissions were measured with a photodiode beamstop. The q calibration was done with a silver behenate sample (Alpha Aeser). Samples were measured in 8–12,900 s frames on a Rigaku HiPix-3000 detector compared to check for beam damage, circular averaged and solvent subtracted by the SAXSLab software (v3.0.2). To analyze the results, Models of the ACE2 fusion protein were initially generated using vmd^34^ and MODELLER^35^. The closed and open structures of CLD and PD from the ACE2-B0AT1 complex^21^ (pdb-id 6m18, pdb-id 6m1d) were combined with a PPC linker and the Fc domain^36^ (pdb-id 4d2n). The linking sequences were added using MODELLER^35^. The model was glycosylated following the pattern proposed by Mehdipour and Hummer^37^ indicated as HT1). The glycans were generated using the glycam web server^38^ and attached to N36, N73, N86, N305, N415, N529, N673, T723, N794 (see Supplementary Table 1). The simulation procedure with the highest possible accuracy (all-atom, explicit water) that is able to capture reasonable time scales of several μs was conducted in order to fit to the SAXS curves. The Amber 20^39^ software suite was used for molecular dynamics simulations. The systems were solvated using tip3p^40^ water in an octahedron water box of 14 Å minimum distance between the protein and the box edge. For the protein ff14SB force field^41^ as used for the glycans GLYCAM_06j-1^42^ parameters were applied. The structures were minimized using 1000 steps of steepest descent. After that the systems were heated up to 300 K (within 16 PS) and equilibrated for 2 ns. We subsequently started two production runs starting from the closed conformation and four productions from the open conformations for 500 ns each. Properties associated to the MD simulations are listed in the Supplementary Table 2. From the three best-fitting models to the SAXS curves we started three simulations for 500 ns from the glycosylated proteins. In addition, we also performed 500 ns production runs from the open and closed conformations with glycosylated proteins. Comparing the observed PD distance and the *R*_*g*_ distribution of the first and the second half of the simulations show that the measurements have equilibrated (Supplementary Fig. 5). The associated restart and coordinate files can be found in https://gitlab.com/TillCyrill/ace2-fc. 6000 individual models were generated using this procedure. Their theoretical SAXS curves were back-calculated using CRYSOL 3.05^43^ and were included for ensemble selection using scripts kindly provided by Dr. Alexander V. Shkumatov. The ensemble selection was performed using GAJOE 3.05^44^. The genetic algorithm was performed in 3000 steps with 100 ensembles per steps and the fit to the SAXS data was limited to a maximum q value of 0.3.

### Circular dichroism (CD)

A Jasco J-1500 spectropolarimeter was used for the CD measurements. The far UV (FUV) spectra and thermal melts were obtained with 0.1 mg/mL ACE2-IgG4-Fc in a 1 mm quartz cuvette. The protein was in phosphate-buffered saline (PBS). The heating rate was 1 K/min.

### Differential scanning calorimetry (DSC)

The samples were measured with a Microcal PEAQ-DSC system (Malvern Panalytical). The temperature ramp was 1 K/min. The ACE2-IgG4-Fc concentration was 1 mg/mL. The sample buffer was PBS. Buffer measurements were subtracted from the thermograms with the protein and processed using the PEAQ-DSC software to determine the melting temperatures.

### Isothermal titration calorimetry (ITC)

A MicroCal PEAQ-ITC (Malvern Panalytical) was used. The ACE2-IgG4-Fc was in the cell, the enzyme inhibitors were in the syringe. The titrations were performed at 25 °C for the interaction with DX600 and at 15 °C for the interaction with MLN4760, because the enthalpy of MLN4760 binding was close to zero at 25 °C. The protein concentration was 10 µM, the inhibitor concentrations were 150–200 µM. For the titration, we used injections of 1.5 and 1.0 µL for the first and second replicate, respectively. The data was analyzed in the PEAQ-ITC software.

### Surface-plasmon resonance (SPR)

A Biacore X-100 system with the Biotin CAPture kit (cytiva) and the HBS-EP + running buffer (cytiva) were used. The RBD of SARS-CoV-2 was purchased with an AviTag (Acrobiosystems) and captured to a level of 100 RU. Increasing concentrations of the ACE2-IgG4-Fc (0.32, 1.6, 8, 40 and 200 nM) were injected over the immobilized RBD in a single-cycle mode. The sensorgrams were evaluated in the Biacore software using the 1:1 binding model.

### Hydrogen-deuterium exchange coupled with mass spectrometry (HDX-MS)

For all H/DX-MS experiments, a fully automated system equipped with a Leap robot (HTS PAL; Leap Technologies, NC), a Waters ACQUITY M-Class UPLC, an H/DX manager (Waters Corp), and a Synapt G2-S mass spectrometer (Waters Corp) were used as previously described^45^ with small modifications. Protein samples with a concentration of 30 µM were diluted in a ratio of 1:20 with 50 mM tris und 150 mM NaCl (pH 7.5) containing deuterium oxide. The samples were incubated with D_2_O for 0 s, 10 s, 1 min, 10 min, 30 min, or 2 h. The exchange was stopped by diluting the labeled protein 1:1 in quenching buffer (200 mM Na_2_HPO_4_ × 2 H_2_O, 200 mM NaH_2_PO_4_ × 2H_2_O, 250 mM Tris (2-carboxyethyl)phosphine, 3 M GdmCl, pH 2.2) at 1 °C. Proteolytic online digestion was performed using an immobilized protease XIII/pepsin (w/w, 1:1) 2.1 × 30 mm column (Novabioassays, LLC) at 20 °C. The resulting peptides were trapped and separated at 0 °C on a Waters AQUITY UPLC BEH C18 column (1.7 mm, 1.0 × 100 mm) by an H_2_O to acetonitrile gradient with both eluents containing 0.1% formic acid (v/v). Eluting peptides were directly subjected to the Synapt TOF mass spectrometer by electrospray ionization. Prior to fragmentation and mass detection, peptides were additionally separated by drift time. Samples were pipetted by a LEAP autosampler (HTS PAL; Leap Technologies, NC). Data analysis was performed with the Waters Protein Lynx Global Server PLGs (version 3.0.3) and the DynamX (Version 3.0) software package. The relative fractional uptake was calculated in DynamX by dividing the deuterium uptake by the number of exchangeable amide hydrogens and presenting the output in percentage.

### Statistics and reproducibility

We have shown the individual measurements from replicates on the graphs. Mean values are calculated where appropriate, standard deviations or 95% confidence intervals are calculated when *n*  ≥  3. Numerical values used for the calculations are provided in the Supplementary Data 1 file. The biophysical data is also confirmed from biological replicates (different batches of protein). The experiments are reproducible.

### Reporting summary

Further information on research design is available in the Nature Portfolio Reporting Summary linked to this article.

## Supplementary information

Supplementary Information

Description of Additional Supplementary Files

Supplementary Data

Reporting Summary

## Data Availability

The MD data is available at 10.5281/zenodo.7716873. The SAXS data was deposited under accession code SASDRU7 at the small angle scattering biological data bank (https://www.sasbdb.org/). Source data for figures can be found in Supplementary Data. Other data are available from the authors upon a reasonable request.
