# Supplementary material for: Extrinsic stabilization of antiviral ACE2-Fc fusion proteins targeting SARS-CoV-2
Source: Commun Biol. 2023 Apr 8;6:386. doi: 10.1038/s42003-023-04762-w (PMC10082628; doi:10.1038/s42003-023-04762-w)
Supplement: Supplementary file 1 — Supplementary Information [file 42003_2023_4762_MOESM1_ESM.pdf]

Supplementary Information

for

Extrinsic stabilization of antiviral ACE2-Fc fusion proteins targeting SARS-CoV-2

Hristo L. Svilenov[1,6*], Florent Delhommel[2,3], Till Siebenmorgen[2,3], Florian Rührnößl[1], Grzegorz M. Popowicz[2,3], Alwin Reiter[4], Michael Sattler[2,3], Carsten Brockmeyer[5] and Johannes Buchner[1*]

[1] Center of Functional Protein Assemblies (CPA) and School of Natural Sciences, Department of Biosciences, Technical University of Munich, Garching, Germany.

[2] Institute of Structural Biology, Helmholtz Zentrum München, Neuherberg, Germany.

[3] Bavarian NMR Center, School of Natural Sciences, Department of Biosciences, Technical University of Munich, Garching, 85748, Munich, Germany.

[4] Formycon AG, Martinsried/Planegg, Germany.

[5] Brockmeyer Biopharma GmbH, Senator-Ernst Str. 2, Marzling, Germany

[6] Current affiliation: Faculty of Pharmaceutical Sciences, Ghent University, Ottergemsesteenweg 460, 9000, Ghent, Belgium

* To whom correspondence should be addressed:

hristo.svilenov@ugent.be, https://orcid.org/0000-0001-5863-9569

johannes.buchner@tum.de, https://orcid.org/0000-0003-1282-7737

phone: +498928913340; fax: +498928913345

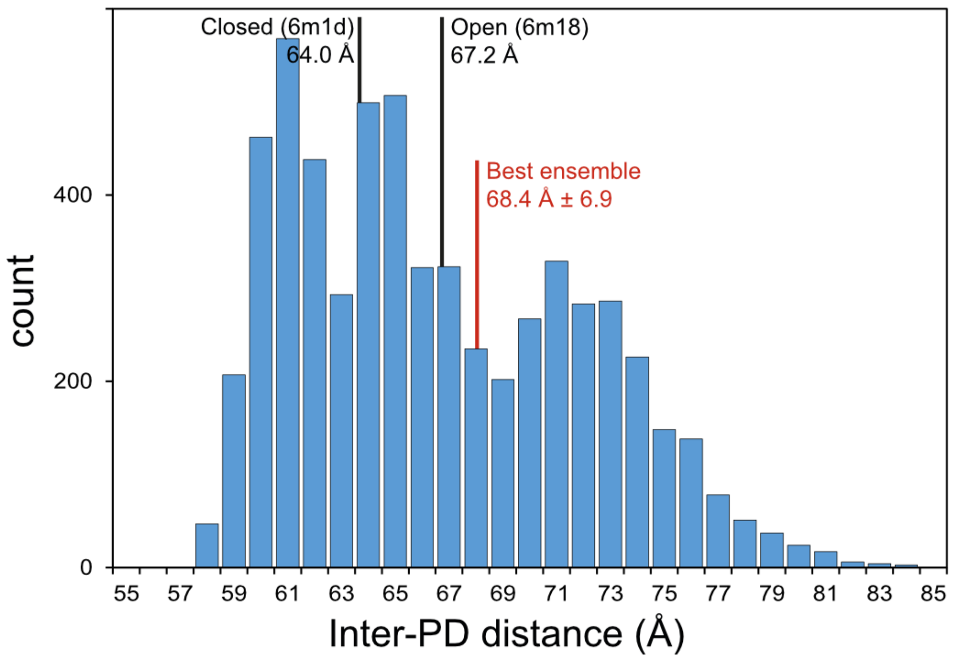

Supplementary Figure 1. Distribution of Inter-PD distance from the pool of all ACE2-Fc conformations generated by MD. This pool was used to select a minimum ensemble fitting best the SAXS data, of which the inter-PD distance is indicated (red), as well as for the known structures of ACE2 in the open and closed conformations.

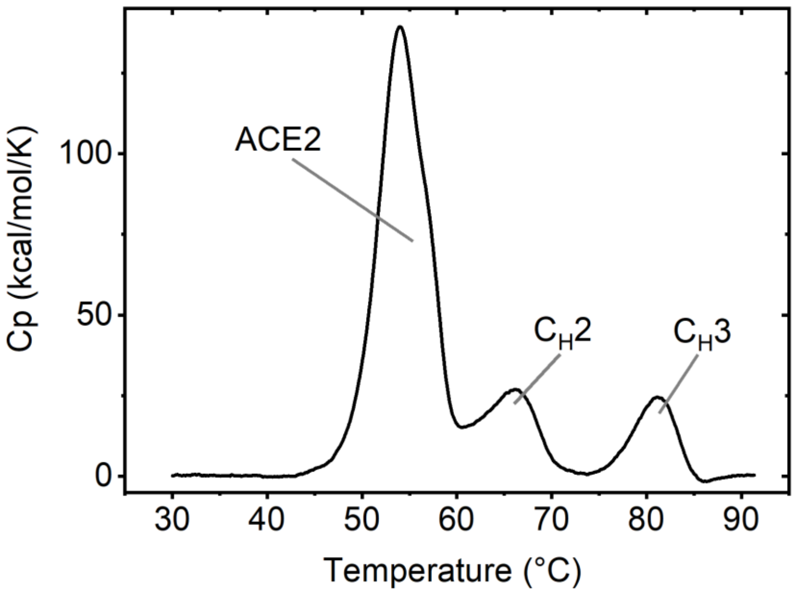

Supplementary Figure 2. Differential scanning microcalorimetry analysis of an ACE2-IgG1-Fc.

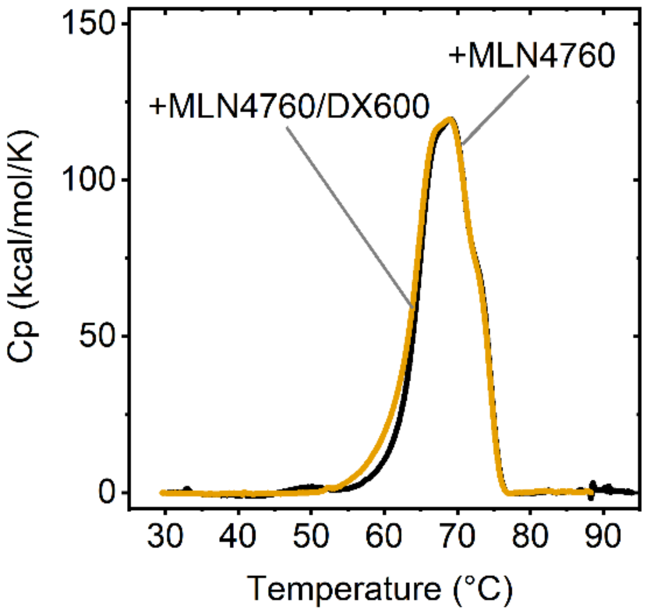

Supplementary Figure 3. DSC analysis of 5 µM ACE2-IgG4-Fc in the presence of 50 µM MLN4760 or 50 µM MLN4760 plus 50 µM DX600

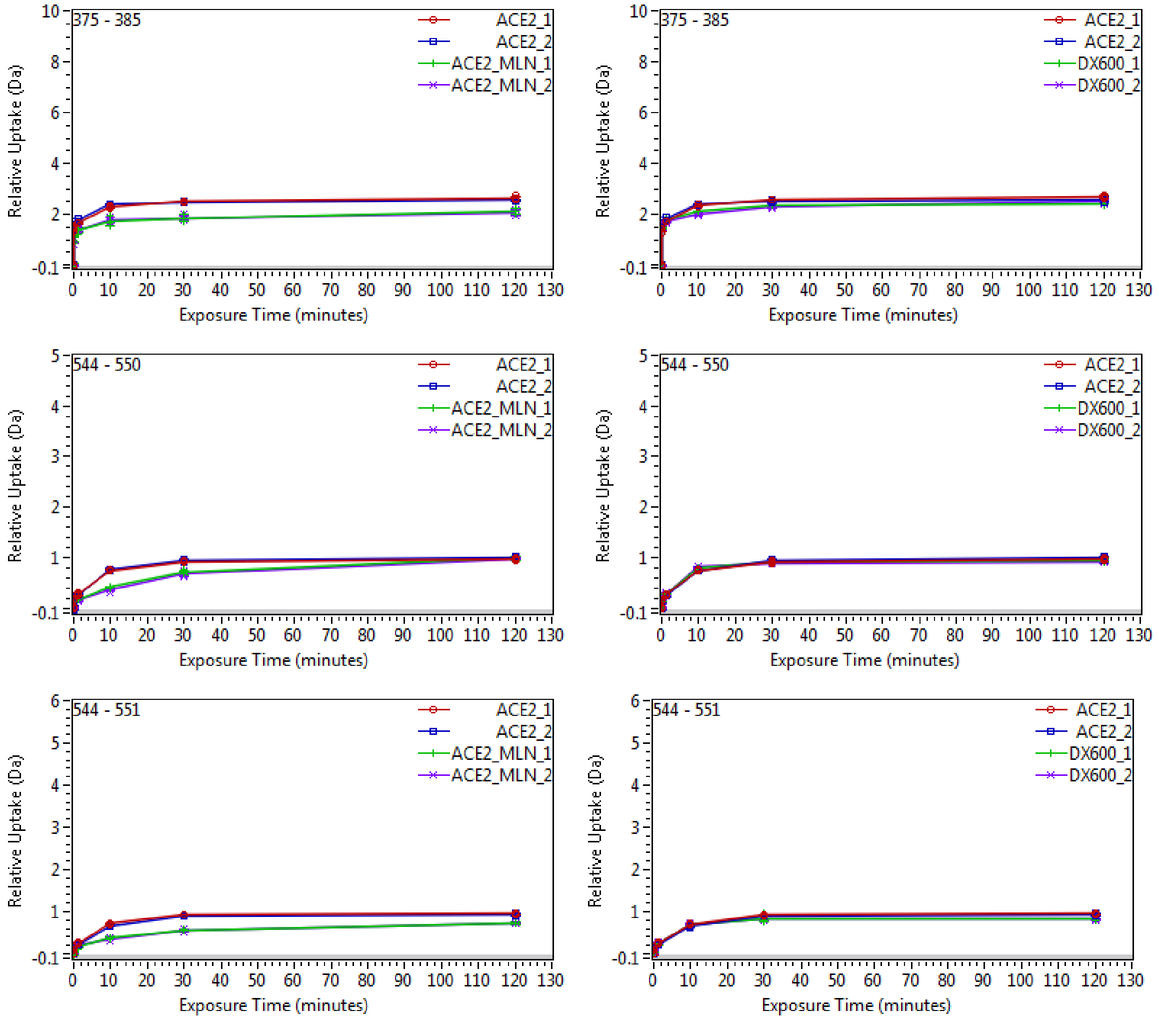

Supplementary Figure 4. Deuterium uptake plots of other peptides in the hinge region between the two subdomains of ACE2 PD. The ACE2 indicates ACE2-IgG4-Fc without inhibitors, ACE2_MLN is ACE2-IgG4-Fc in the presence of MLN4760, DX600 is ACE2-IgG4-Fc in the presence of DX600.

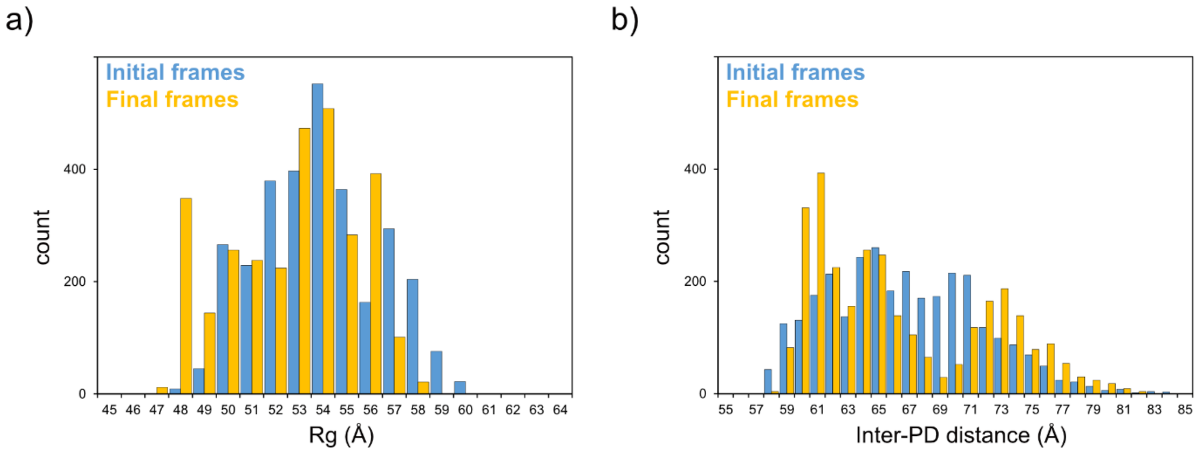

Supplementary Figure 5. Comparison of the first (blue) and the final (yellow) half of the MD simulation runs. **a)** R_g distribution and **b)** inter-PD distance distribution show the same general trend in both subsets.

Supplementary Table 1. Overview of the attached glycans and the corresponding protein sites.

Protein Site	Glycan
N36,N529	DGlcpNAcb1-4DGlcpNAcb1-2DManpa1-6[DGlcpNAcb1-2DManpa1-3]DManpb1-4DGlcpNAcb1-4DGlcpNAca1-OH
N73, N673	DGlcpNAcb1-2DManpa1-6[DGalpb1-4DGlcpNAcb1-2DManpa1-3]DManpb1-4DGlcpNAcb1-4[LFucpa1-3]DGlcpNAca1-OH
N86, N415	DGlcpNAcb1-2DManpa1-6[DGlcpNAcb1-2DManpa1-3]DManpb1-4DGlcpNAcb1-4[LFucpa1-3]DGlcpNAca1-OH
N305	DGlcpNAcb1-2DManpa1-6[DGlcpNAcb1-4[DGlcpNAcb1-4DGlcpNAcb1-2]DManpa1-3]DManpb1-4DGlcpNAcb1-4DGlcpNAca1-OH
T723	DNeup5Aca2-6[DNeup5Aca2-3DGalpb1-3]DGalpNAca1-OH
N794	DGlcpNAcb1-2DManpb1-6DManpb1-4DGlcpNAcb1-4[LFucpa1-6]DGlcpNAca1-OH

Supplementary Table 2. Overview of the properties and associated values used for the MD simulations.

Property	Value
Simulation box dimensions	14 Å minimum distance to box edge
Total number of atoms glycosylated	593412
Total number of water molecules glycosylated	186666
Total number of ions	58
Thermostat	Langevin
Collision frequency in ps^{-1}	2
Protein force field	FF14SB
Glycan force field	GLYCAM_06j-1
Water force field	TIP3P
Nonbonded cutoff	8.5 Å
