# Supplementary material for: Extrinsic stabilization of antiviral ACE2-Fc fusion proteins targeting SARS-CoV-2
Source: Commun Biol. 2023 Apr 8;6:386. doi: 10.1038/s42003-023-04762-w (PMC10082628; doi:10.1038/s42003-023-04762-w)
Supplement: Supplementary file 2 — Description of Additional Supplementary Files [file 42003_2023_4762_MOESM2_ESM.pdf]

Description of Additional Supplementary Files

File name: Supplementary Data 1

Description: The source data behind the graphs in the paper
